# Vascular risk factors mediate the relationship between education and white matter hyperintensities

**DOI:** 10.1002/alz.70972

**Published:** 2025-12-15

**Authors:** Shima Raeesi, Yashar Zeighami, Roqaie Moqadam, Cassandra Morrison, Mahsa Dadar

**Affiliations:** ^1^ Cerebral Imaging Center Douglas Research Center Montreal Quebec Canada; ^2^ Department of Psychiatry McGill University Montreal Quebec Canada; ^3^ Department of Medicine University of Montreal Montreal Quebec Canada; ^4^ Department of Psychology Carleton University Ontario Canada

**Keywords:** Alzheimer's disease, cognitive decline, cognitive reserve, education, vascular risk factors, white matter hyperintensities

## Abstract

**INTRODUCTION:**

Education can protect against cognitive decline and dementia through cognitive reserve and reduced vascular risk. This study examined whether vascular risk mediated the relationship between education and white matter hyperintensity (WMH) burden.

**METHODS:**

Data from 1443 older adults from the National Alzheimer's Coordinating Center were analyzed. A composite vascular score was created using diabetes, hypertension, hypercholesterolemia, smoking, alcohol abuse, body mass index, and blood pressure. Linear regressions and mediation analyses examined associations and indirect effects between education, vascular risk, and WMHs, adjusting for age, sex, and diagnosis.

**RESULTS:**

Higher education was associated with lower vascular risk (*p* < 0.001) and WMH burden (*p* = 0.004). Mediation analysis showed an indirect effect of education on WMH via vascular risk (a*b = –0.02, *p* < 0.001), accounting for 27% of the total effect.

**DISCUSSION:**

Education influences cerebrovascular health by reducing vascular risk. Addressing vascular health may reduce WMH burden.

**Highlights:**

- Education is associated with lower WMH burden in aging adults.
- Vascular risk factors mediate the education–WMH relationship.
- Higher education predicts better vascular profiles and less WMH accumulation.

## 1 BACKGROUND

White matter hyperintensities (WMHs) are magnetic resonance imaging (MRI)‐detectable lesions indicative of cerebrovascular disease.^1^ Their presence is strongly associated with cognitive decline and an increased risk of dementia, including Alzheimer's disease (AD).^2^, ^3^, ^4^, ^5^, ^6^ WMHs often present as hyperintense regions on T2‐weighted and fluid‐attenuated inversion recovery (FLAIR) MRI scans and are thought to reflect chronic ischemia and microvascular damage.^3^, ^6^ WMHs are particularly common among older adults, increasing in prevalence with age^3^, ^7^, ^8^, ^9^ and becoming more extensive in individuals with vascular comorbidities, such as hypertension and diabetes.^10^, ^11^, ^12^, ^13^

WMHs are increasingly recognized as a biomarker of cerebral small vessel disease; however, the pathophysiology of WMHs is complex and multifactorial, involving chronic hypoperfusion, endothelial dysfunction, and small vessel pathology.^6^, ^14^ Evidence from neuropathological studies indicates that WMHs may arise from a combination of ischemic demyelination, gliosis, and axonal loss.^15^, ^16^ Some studies suggest that WMHs may represent a continuum of cerebrovascular injury, ranging from mild, asymptomatic changes to extensive, clinically significant damage. WMH burden is also predictive of future cognitive decline, progression to mild cognitive impairment (MCI), and an increased risk of dementia.^8^, ^17^, ^18^ Finally, the spatial distribution of WMHs has been shown to relate differently to dementia and vascular risk factors. For example, frontal WMHs are more closely linked to vascular risk factors, whereas parietal WHMs are more strongly linked to dementia risk and conversion.^15^, ^19^

Education has long been recognized as a protective factor against cognitive decline, a concept often attributed to the cognitive reserve hypothesis.^20^, ^21^, ^22^, ^23^ This hypothesis posits that individuals with higher educational attainment develop more robust cognitive processes or neural networks, allowing them to better cope with age‐related brain changes and pathology compared to people with lower education.^23^, ^24^, ^25^ However, the mechanisms through which education confers this protective effect are multifaceted and not entirely understood. While some studies emphasize the role of lifelong cognitive engagement, others highlight the potential for education to modify health behaviors, thereby reducing vascular risk.^24^, ^26^, ^27^ Emerging evidence suggests that education may influence health behaviors and lifestyle choices, leading to reduced exposure to vascular risk factors.^27^, ^28^, ^29^ For instance, individuals with higher education levels are more likely to engage in health‐promoting behaviors, have better access to healthcare resources, and possess greater health literacy, all of which contribute to lower incidences of different vascular conditions.^27^, ^28^, ^29^ These factors may collectively reduce the burden of WMHs and subsequent cognitive decline.^15^ High educational attainment is linked to better cardiovascular health, both of which are critical in reducing cerebrovascular damage. Individuals with higher education demonstrate slower WMH progression over time, even when controlling for age and vascular risk factors.^29^, ^30^

There is increasing interest in the potential mediation effect of vascular risk factors on the relationship between education and WMH burden. Understanding the interplay between education, vascular risk, and WMHs is crucial, as it underscores the potential of educational interventions and public health strategies aimed at reducing vascular risks to mitigate WMH accumulation and preserve cognitive function.^15^, ^20^, ^21^ Given the increasing prevalence of dementia and cerebrovascular diseases, identifying modifiable factors that influence WMH progression remains a public health priority.^20^, ^22^ While education appears to offer a protective effect against cognitive decline, this benefit is likely influenced by a complex interplay of factors, including vascular health. Investigating the mediating role of vascular risk factors in the relationship between education and WMH burden can provide valuable insights into preventive strategies for cognitive impairment and dementia.^22^

The primary objective of this study was to investigate whether vascular risk factors mediated the relationship between education and WMH volume. Data were drawn from the National Alzheimer's Coordinating Center (NACC, https://naccdata.org/) dataset,^31^ which includes comprehensive clinical, MRI, and neuropsychological assessments from multiple sites across the United States, providing a robust, community‐based representation of aging and cognitive health.

**RESEARCH IN CONTEXT**

1. **Systematic review**: The authors reviewed the literature using traditional sources (e.g., PUBMED) and observed that, while there are numerous reports examining the relationship between WMHs, risk factors, cognitive decline, and dementia, few studies have examined how education influences WMHs.
2. **Interpretation**: Our findings suggest that education directly (and indirectly) influences WMH burden. That is, higher education is associated with lower rates of vascular risk factors, and vascular risk mediates the relationship between education and WMHs.
3. **Future directions**: Future research should investigate these associations in racially and ethnically diverse samples with sufficient statistical power.

## 2 METHODS

### 2.1 National Alzheimer's Coordinating Center participants

Data were obtained from the NACC database, including the NACC Uniform Data Set (UDS) and MRI Data Set, with T1‐weighted and FLAIR MRI scans available to extract regional WMH measures.^31^, ^32^, ^33^ Baseline data from individuals aged 55 and over with diagnoses of cognitively normal, amnestic MCI, non‐amnestic MCI, and AD were included in the study. Participants were selected based on the availability of MRI data and vascular risk factor information. Cognitive diagnoses were derived using the NACCTMCI variable (clinician‐assigned MCI diagnosis) and the NACCETPR variable (representing the primary diagnosis) as well as Clinical Dementia Rating‐Sum of Boxes (CDR_SB) scores, ensuring consistency in cognitive categorization. Participants with CDR_SB of 0 and no neurological disorders based on NACCETPR variable were classified as “NC,” those with a CDR_SB between 0.5 and 4 were classified as “MCI,” and those with CDR_SB > 4.5 were classified as “AD.” Participants with other neurological disorders (e.g., Parkinson's disease, Lewy body dementia, frontotemporal dementia) or inconsistent CDR scores and diagnostic labels (e.g., individuals with CDR_SB > 4.5 and no disorders identified based on NACCETPR) were excluded. Education was measured as a continuous variable representing the total number of years of formal schooling completed.

### 2.2 MRI and volumetric WMH measurements

**FIGURE 1:**
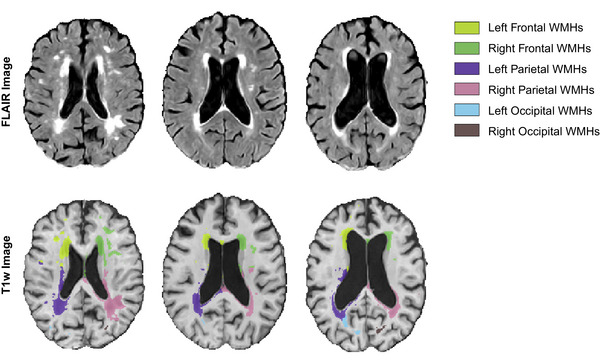
Illustrative examples of WMH segmentations derived from FLAIR and T1‐weighted MRI scans across three participants with varying WMH burdens. FLAIR, fluid‐attenuated inversion recovery MRI scans; MRI, magnetic resonance imaging; WMH, white matter hyperintensity.

MRI preprocessing: T1‐weighted and FLAIR MRI scans were processed using an open‐source image processing pipeline, integrating tools from the MINC Toolkit version 2 (MINC Toolkit) and Advanced Normalization Tools (ANTs) Toolkit.^34^ The preprocessing pipeline included essential steps such as noise reduction,^35^ correction for intensity non‐uniformity,^36^ and intensity normalization to a standardized range of 0 to 100. After initial preprocessing, the T1‐weighted images underwent linear registration^37^ to the MNI‐ICBM152 (Montreal Neurological Institute ‐ International Consortium for Brain Mapping 152) average template^38^ using nine parameters (translation, rotation, and scaling) to correct for head size and orientation differences, followed by a non‐linear registration.^39^, ^40^ WMH volumes were identified based on T1‐weighted and FLAIR images using BISON,^41^ an automated segmentation tool previously validated for WMH detection in other multicenter studies^42^ as well as in NACC.^4^, ^43^, ^44^, ^45^ This method leverages a random forest classifier combined with both location‐specific features and intensity features, which are derived from a comprehensive library of manually segmented scans. All processing steps, including linear and non‐linear registration and WMH segmentation, were visually quality controlled by an experienced rater (RM), blind to clinical diagnosis. Quality control primarily aimed to ensure accurate and precise registration to the MNI template and correct delineation of WMH masks. Participants were excluded if they had significant segmentation errors (e.g., over‐ or under‐labeling of WMHs, usually occurring due to the presence of motion or other artifacts) or if registration failed, indicated by misaligned cortical or ventricular boundaries. Using these exclusion criteria, 28 participants were removed from the analyses. WMH volume was quantified as the number of voxels labeled as WMH within the standard space (cubic millimeters [mm^3^]), that is, normalized for intracranial volume. Regional WMH volumes were calculated separately for each brain lobe (frontal, temporal, parietal, and occipital) and hemisphere using the Hammer's lobar atlas.^46^, ^47^ Figure 1 shows an example of the WMH segmentations for three NACC participants. To achieve a normal distribution, the calculated WMH volumes were log‐transformed prior to statistical analysis.

### 2.3 Vascular risk factors

To comprehensively evaluate the influence of vascular risk factors on WMH burden, we systematically assessed multiple variables reflecting vascular health. Diabetes status was determined using values from the DIABET and DIABETES variables based on health history. Hypertension was identified using the HYPERT and HYPERTEN variables. Hypercholesterolemia was derived from the HYPERCHO and HYPERCHOL variables. Alcohol abuse was assessed using the ALCOHOL variable, which was defined as alcohol abuse causing clinically significant impairment in areas such as work, driving, legal, or social domains over a 12‐month period. For all these variables, binary coding was used, where 0 indicated the absence and 1 indicated the presence of the risk factor.

In contrast, the following vascular indicators were treated as continuous variables: body mass index (BMI), calculated using the NACCBMI column based on recorded height and weight; systolic and diastolic blood pressure, derived from the BPSYS and BPDIAS columns, respectively; and smoking behavior, which was quantified using total years smoked (SMOKYRS) and average number of packs smoked per day (PACKSPER). These continuous variables allowed for more granular assessment of vascular risk burden and were analyzed accordingly.

### 2.4 Vascular composite score

To facilitate mediation analysis and assess the cumulative impact of vascular risk factors, we developed a vascular composite score.^10^, ^16^, ^48^, ^49^, ^50^ This score enabled evaluation of whether the combined vascular risk mediated the relationship between education and WMHs. To achieve uniform integration within the composite score, continuous variables such as BMI, systolic blood pressure, and diastolic blood pressure were transformed into binary variables. Following the guidelines from the National Institutes of Health and the National Institute on Aging for older adults, as well as the 2017 American College of Cardiology and the American Heart Association Guideline for the Prevention, Detection, Evaluation, and Management of High Blood Pressure in Adults, high systolic blood pressure was defined as ≥130 mmHg and high diastolic blood pressure as ≥80 mmHg.^51^, ^52^ Based on the Centers for Disease Control and Prevention, a BMI of 25 or more is categorized as high (overweight or obesity).^26^ Consequently, for these three variables, a value of 1 indicates elevated levels, while 0 indicates normal levels. Smoking status was also incorporated into the composite score, with individuals who had any history of smoking coded as 1 and non‐smokers coded as 0. The vascular composite score was then calculated by summing the presence (1) or absence (0) of the eight binary‐coded vascular risk factors (diabetes, hypertension, hypercholesterolemia, smoking history, alcohol abuse, high BMI, high systolic, and high diastolic blood pressure), resulting in a continuous measure ranging from 0 to 8 per participant. This additive score provides a transparent and interpretable index of cumulative vascular burden, particularly appropriate for population‐based datasets such as NACC that lack detailed continuous biomarkers (e.g., lipid subfractions or inflammatory markers). Equal weighting also potentially reduces overfitting and avoids data‐driven weighting, thereby preserving the contribution of each clinically meaningful factor.

In addition to the unweighted composite score, we computed a weighted Atherosclerotic Cardiovascular Disease (ASCVD) BMI‐based score following published guidelines.^53^, ^54^, ^55^ The ASCVD BMI‐based model uses age, sex, race, systolic blood pressure, antihypertensive treatment, diabetes, smoking status, and BMI to estimate the 10‐year risk of ASCVD. A higher score indicates a greater risk for future cardiovascular events. Unlike the traditional ASCVD risk equations, which require lipid profile data, the BMI‐based version substitutes BMI for total and high‐density lipoprotein cholesterol. Because lipid measures were not available in the NACC dataset, the BMI‐based version was selected to enable estimation of risk. However, this score also has important limitations: It was originally developed for adults aged 30 to 74 years who were free of clinical cardiovascular disease at baseline, conditions not strictly met in our cohort of older adults with variable and sometimes unknown vascular histories. For these reasons, we initially did not rely on the ASCVD score as our primary index but included it as a sensitivity analysis to evaluate the robustness of our mediation findings under a weighted clinical risk model. Pearson's correlation analysis demonstrated that the unweighted composite score and the ASCVD BMI‐based score were moderately correlated (*r* = 0.33, 95% CI [0.28 to 0.38], *p* < 0.001), both in raw and *z*‐scored versions, suggesting that the two indices capture overlapping yet partially distinct aspects of vascular burden.

### 2.5 Statistical analysis

Demographic and clinical characteristics were analyzed using independent sample *t* tests and chi‐squared tests, with false discovery rate (FDR) correction applied for multiple comparisons.^56^ All analyses were conducted in MATLAB R2024b and R (version 4.3). To investigate the relationship between education and individual vascular risk factors, we employed a series of linear regression models, adjusting for covariates including age, sex, race, and diagnostic status. Vascular risk factors were modeled based on their measurement level: categorical variables (e.g., presence or absence of diabetes, hypertension, hypercholesterolemia, and alcohol abuse) were treated as binary outcomes, while continuous variables (e.g., BMI, systolic and diastolic blood pressure, smoking years, packs per day, and pack‐years) were treated as continuous outcomes. Accordingly, linear regression was used for continuous outcomes, and logistic regression was applied to binary outcomes to ensure appropriate modeling for each variable type.

In both approaches, continuous predictors were *z*‐scored prior to analysis to standardize their distributions and facilitate interpretability. Each vascular risk factor was analyzed independently to estimate its association with education. The model equation for continuous outcomes can be expressed as

(1) VascularRiskFactor∼Education+Age+Sex+Race+Diagnosis

Effect sizes (estimates) were computed to assess whether the observed associations differed significantly from zero. Additional analyses were conducted to evaluate the relationship between education and WMH volume. WMH volumes were log‐transformed to achieve a more normal distribution and *z*‐scored prior to the analyses. This analysis was completed for each region of interest, including total, right and left frontal, temporal, parietal, and occipital. Thus, the primary dependent variable was the log‐transformed WMH volume, while the independent variable of interest was education.

(2) WMHVolume∼Education+Age+Sex+Race+Diagnosis

To investigate the mediating effect of vascular risk factors on the relationship between education and WMH burden, a mediation model was estimated using the structural equation modeling (SEM) approach in the mediation package in R.^57^ Despite the cross‐sectional nature of this analysis, temporal precedence is conceptually supported: Education occurs over a lifetime and precedes both vascular risk and WMH burden, while vascular risk factors typically arise earlier in life and progress over time. WMHs, reflecting cumulative cerebrovascular damage, were measured at the time of the MRI visit. Therefore, even though the cross‐sectional design still limits causal inference, the assumed ordering (education → vascular risk → WMH) is biologically and temporally plausible. All variables were standardized (*z*‐scored) before inclusion. Age, sex, race, and diagnosis were included as covariates. To assess the significance of the indirect effects, bootstrapping was performed with 1000 resamples. The proportion of the total effect mediated was calculated to assess the relative contribution of the indirect pathway compared to the direct effect.

All regression and mediation analyses were performed using both the unweighted composite vascular risk score and the weighted ASCVD BMI‐based score as mediators to ensure robustness of results across scoring methods. Interaction terms between education and race were added to both the mediator and outcome models, with White participants set as the reference group, allowing us to test whether associations between education, vascular risk, and WMH burden differed significantly across racial categories.

(3) VascularRiskFactor∼Education×Race+Age+Sex+Diagnosis

(4) $$\begin{array}{ccc} \mathrm{WMH}\mathrm{Volume}\;∼\;\mathrm{Education}\;\times & & \;\mathrm{Race}+\mathrm{Vascular}\mathrm{Risk}\mathrm{Factor}\;\times \;\mathrm{Race}\\ + & & \mathrm{Age}+\mathrm{Sex}+\mathrm{Race}+\mathrm{Diagnosis} \end{array}$$

In addition, stratified mediation models were performed within each race category to provide descriptive comparisons. These included:

1. Mediation in the full sample with race category included as a covariate, where the model was conditioned on race while adjusting for all covariates; and
2. Stratified (within‐race) mediation, where the mediation analysis was repeated separately within each race group.

Single‐mediator models were estimated for individual continuous vascular risk factors (BMI, systolic and diastolic blood pressure, and years and packs of smoking) to identify which specific factors contributed most to the mediation effect. Sensitivity analyses were also performed, including leave‐one‐out models in which one vascular risk factor was excluded at a time to assess the robustness of the results. Statistical significance was defined as FDR‐adjusted^56^ *p* < 0.05 to account for multiple comparisons. FDR correction was applied across total and regional WMH mediation models, as well as all sensitivity analyses, and all *p* values reported in the text reflect FDR‐adjusted values.

## 3 RESULTS

### 3.1 Demographics and clinical data

Table 1 summarizes the characteristics of study participants. The total sample included 1443 older adults (1089 White, 143 Black, 171 Hispanic, and 40 Asian). The mean age of the full sample was 74.3 ± 8.6 years, with 59% female. Age differed significantly across racial groups: White participants (73.9 ± 8.5 years) were slightly younger than Black (76.0 ± 8.9 years, *p* = 0.02) and Asian participants (77.4 ± 8.8 years, *p* = 0.03), whereas age differences among all other racial groups were not significant (all *p* > 0.05). Sex distribution differed across races (*p* = 0.04), driven by a higher proportion of females among Black participants (69%) compared to White participants (57%), while other between‐group differences were non‐significant. Education also differed markedly across racial groups. White participants had the highest mean education (15.9 ± 2.9 years), followed by Asian (15.2 ± 3.0 years) and Black (14.3 ± 3.4 years) participants, whereas Hispanic participants had substantially lower education levels (10.6 ± 4.9 years; all *p* < 0.001 compared with other groups).

**TABLE 1:** Demographic, clinical, and neuroimaging characteristics for study participants.

	Total	White	Black	Hispanic	Asian	Group differences
Total number	1443	1089	143	171	40	N/A
Age (mean, SD)	74.29 (8.57)	73.86 (8.49)	76.04 (8.88)	74.87 (8.47)	77.40 (8.77)	W versus B: 0.02, W versus A: 0.03
Female (*n*, %)	852 (59)	620 (57)	99 (69)	108 (63)	25 (62)	W versus B: 0.04
Education (years, mean, SD)	15.08 (3.67)	15.87 (2.86)	14.32 (3.38)	10.64 (4.93)	15.25 (2.99)	W versus B < 0.001, W versus H < 0.001, B versus H < 0.001, A versus H < 0.001
Diabetes (*n*, %)	230 (16)	110 (10)	54 (38)	54 (31)	12 (30)	W versus B < 0.001, W versus H < 0.001, W versus A < 0.001
Hypertension (*n*, %)	686 (47)	448 (41.14)	101 (71)	114 (67)	23 (57)	W versus B < 0.001, W versus H < 0.001
Hypercholesterolemia (*n*, %)	725 (50)	521 (47.84)	84 (59)	96 (56)	24 (60)	–
Alcohol abuse (*n*, %)	67 (5)	46 (4.22)	5 (3)	15 (9)	1 (2)	–
Smoking (*n*, %)	576 (40)	430 (39)	63 (44)	74 (43)	9 (22)	–
High BMI (≥25 kg/m^2^) (*n*, %)	862 (60)	619 (57)	101 (71)	127 (74)	15 (37)	W versus B: 0.003, W versus H < 0.001, W versus A: 0.03, A versus B < 0.001, A versus H < 0.001
High systolic BP (≥130 mmHg) (*n*, %)	873 60)	626 (57)	103 (72)	119 (69)	25 (62)	W versus B: 0.007, W versus H: 0.01
High diastolic BP (≥80 mmHg) (*n*, %)	512 (35)	379 (35)	59 (41)	63 (37)	11 (21)	–
Composite vascular score (mean, SD)	2.71 (1.54)	2.50 (1.47)	3.40 (1.55)	3.29 (1.55)	2.75 (1.60)	W versus B < 0.001, W versus H < 0.001, A versus B: 0.007, A versus H: 0.02
ASCVD BMI‐based score (mean, SD)	26.94 (1.26)	26.79 (1.23)	27.25 (1.18)	27.68 (1.27)	27.12 (1.44)	W versus B < 0.001, W versus H < 0.001, B versus H: 0.004, A versus H: 0.02
Total WMH (cm^3^) (mean, SD)	19.39 (19.13)	18.09 (18.57)	27.23 (22.98)	19.81 (17.96)	24.94 (16.66)	W versus B < 0.001, B versus H: 0.002

*Note*: Values are presented as total number and percentage or mean and standard deviation. Diabetes, hypertension, hypercholesterolemia, alcohol abuse, and smoking were reported as 1 (has the condition) or 0 (does not have the condition).

Abbreviations: A, Asian; ASCVD, Atherosclerotic Cardiovascular Disease risk assessment; B, Black; BMI, body mass index; BP, blood pressure; H, Hispanic; SD, standard deviation; W, White; WMH, white matter hyperintensity.

Prevalence of vascular risk factors was high across racial groups. Diabetes was present in 16%, hypertension in 47%, and hypercholesterolemia in 50% of participants. Alcohol abuse was relatively uncommon (5%), while smoking history was reported by 40% of the sample. Diabetes showed significant racial differences, with all non‐White groups having a higher prevalence than White participants. In particular, the prevalence of diabetes was significantly higher among Black (38%), Hispanic (31%), and Asian (30%) participants than among White participants (10%; all *p* < 0.001). No significant differences were observed among the non‐White groups themselves (*p* > 0.05). Hypertension prevalence also differed significantly, being highest in Black (71%) and Hispanic (67%) participants compared with White participants (41%, both *p* < 0.001); no difference was found between other groups (*p* > 0.05). In contrast, hypercholesterolemia, alcohol abuse, smoking, and high diastolic blood pressure (≥80 mmHg) did not differ significantly between racial groups (all *p* > 0.05). High systolic blood pressure (≥130 mmHg) was more frequent among Black (72%) and Hispanic (69%) participants compared with White participants (57%, *p* = 0.007 and *p* = 0.01, respectively). High BMI (≥25 kg/m^2^) also differed significantly across races, with Hispanic (74%) and Black (71%) participants showing the highest prevalence, followed by White (57%) and Asian (37%). Asian participants had significantly lower prevalence of high BMI compared with all other groups (vs Black and Hispanic, *p* < 0.001; vs White, *p* = 0.03), while both Black and Hispanic participants had higher prevalence than White participants (*p* = 0.003 and *p* < 0.001, respectively). No significant difference was found between Black and Hispanic participants (*p* = 0.67).

The mean composite vascular score was 2.71 ± 1.54 (out of 8 possible points), indicating a moderate cumulative vascular risk. Scores were highest among Black (3.40 ± 1.55) and Hispanic (3.29 ± 1.55) participants, followed by Asian (2.75 ± 1.60) and White (2.50 ± 1.47) participants. Black participants had significantly higher composite scores than both White (*p* < 0.001) and Asian (*p* = 0.007) participants, while Hispanic participants had higher scores than White (*p* < 0.001) and Asian (*p* = 0.02) groups. No significant difference was found between Black and Hispanic participants (*p* > 0.05). The mean ASCVD BMI‐based score for the total sample was 26.94 ± 1.26, reflecting a moderate overall vascular risk. Hispanic (27.68 ± 1.27) and Black (27.25 ± 1.18) participants had the highest scores across all racial groups, followed by Asian (27.12 ± 1.44) and White (26.79 ± 1.23) individuals, suggesting slightly higher vascular risk profiles among non‐White participants. The ASCVD scores of Black participants were significantly higher than those of White (*p* < 0.001) and Hispanic participants (*p* = 0.004), while Hispanic participants scored higher in both White (*p* < 0.001) and Asian (*p* = 0.02) groups. No significant difference was observed between Asian and White participants (*p* > 0.05).

The mean total WMH volume for the full cohort was 19,393 mm^3^ (SD = 19,131). Black participants exhibited the highest WMH burden (27,234 ± 22,987 mm^3^), followed by Asian (24,946 ± 16,662 mm^3^), Hispanic (19,818 ± 17,964 mm^3^), and White (18,093 ± 18,570 mm^3^) participants. Black participants had significantly greater WMH burden than both White (*p* < 0.001) and Hispanic (*p* = 0.002) participants, while differences among all other racial groups were not statistically significant (*p* > 0.05).

### 3.2 Education and vascular risk factors

Higher educational attainment was significantly associated with lower vascular risk across multiple domains (Table 2). Specifically, education showed significant inverse associations with diabetes (*β* = –0.25), hypertension (*β* = –0.26), systolic blood pressure (*β* = –0.09), hypercholesterolemia (*β* = –0.34), alcohol abuse (*β* = –0.18), smoking years (*β* = –0.13), smoking packs per day (*β* = –0.09), and BMI (*β* = –0.18) (all *p* < 0.001). No significant association was observed for diastolic blood pressure (*β* = 0.03, *p* = 0.30).

**TABLE 2:** Associations between educational attainment and vascular risk factors.

	Total			White			Black			Hispanic			Asian		
	Estimate	tStat	*P* value	Estimate	tStat	*P* value	Estimate	tStat	*P* value	Estimate	tStat	*P* value	Estimate	tStat	*P* value
Diabetes	−0.25	−5.742	<0.001	−0.12	−2.68	0.008	−0.19	−1.11	0.37	−0.28	−2.36	0.05	−0.32	−0.85	0.68
Hypertension	−0.26	−5.777	<0.001	−0.15	−2.96	0.004	−0.21	−1.44	0.35	−0.31	−2.68	0.03	−0.18	−0.56	0.72
Systolic blood pressure	−0.09	−3.41	<0.001	−0.09	−3.07	0.003	0.08	−0.95	0.34	−0.11	−1.43	0.18	−0.17	−0.96	0.68
Diastolic blood pressure	0.03	1.07	0.30	0.03	−1.03	0.30	0.01	1.10	0.27	0.05	0.69	0.49	−0.14	−0.83	0.68
Hypercholesterolemia	−0.34	−7.15	<0.001	−0.16	−3.02	0.003	−0.36	−2.10	0.24	−0.69	−4.68	<0.001	−0.04	−0.14	0.72
Alcohol abuse	−0.18	−5.15	<0.001	−0.11	−2.62	0.07	−0.17	−1.49	0.34	−0.24	−3.36	<0.001	−0.16	−1.01	0.68
Smoking (years)	−0.13	−4.692	<0.001	−0.16	−4.83	<0.001	−0.13	−1.46	0.35	−0.16	−2.08	0.06	0.08	0.45	0.72
Smoking (packs per day)	−0.09	−3.273	0.001	−0.16	−4.94	<0.001	−0.09	−0.99	0.33	−0.08	−1.00	0.35	−0.06	−0.31	0.76
BMI	−0.18	−7.13	<0.001	−0.14	−4.65	<0.001	−0.12	−1.34	0.38	−0.17	−2.17	0.06	−0.17	−0.98	0.68
Composite score	−0.18	−9.347	<0.001	−0.19	−6.46	<0.001	−0.11	−1.24	0.32	−0.12	−1.48	0.18	−0.43	−2.57	0.10
ASCVD score	−0.11	−6.32	<0.001	−0.09	−5.37	<0.001	−0.11	−2.00	0.05	−0.14	−2.71	0.009	−0.24	−2.82	0.009

*Note*: Bolded values are those that remained significant after correction for multiple comparisons.

Abbreviations: ASCVD, Atherosclerotic Cardiovascular Disease risk assessment; BMI, body mass index.

Both the composite vascular score (*β* = –0.18) and the ASCVD BMI‐based score (*β* = –0.11) were inversely associated with education (both *p* < 0.001), indicating that individuals with higher educational attainment had a lower cumulative vascular risk burden. When stratified by race, the inverse association between education and vascular risk remained consistent in White participants, with significant effects observed for diabetes, hypertension, systolic blood pressure, hypercholesterolemia, smoking, BMI, and both composite and ASCVD BMI‐based scores (all *p* < 0.01). Associations were mostly nonsignificant in non‐White groups, likely due to their smaller sample sizes and limited power.

### 3.3 Education and WMH burden

Linear regression analyses revealed that higher educational attainment was significantly associated with lower total WMH burden (*β* = –0.05, *p* = 0.004) after adjusting for key covariates, including age, sex, race, and cognitive status, indicating that education contributes independently to cerebrovascular health. Region‐specific analyses further demonstrated that education was inversely associated with WMH volume across all lobes (all *p* < 0.05), although the magnitude of these associations varied by region (Table 3). The strongest effects were observed in the parietal and occipital regions, followed by the temporal and frontal lobes, suggesting that education‐related differences in WMH burden are widespread but regionally heterogeneous. In race‐stratified models, the negative association between education and total WMH burden (*β* = –0.07, *p* = 0.01), as well as several regional WMH measures, remained significant only among White participants.

**TABLE 3:** Associations between education and total and regional WMH volumes.

	Total			White			Black			Hispanic			Asian		
	Estimate	tStat	*P* value	Estimate	tStat	*P* value	Estimate	tStat	*P* value	Estimate	tStat	*P* value	Estimate	tStat	*P* value
Total WMH	−0.05	−3.060	0.004	−0.07	−2.66	0.01	−0.10	−1.22	0.33	−0.08	−1.38	0.22	0.02	0.15	0.87
L frontal	−0.05	−2.136	0.03	−0.05	−1.94	0.05	−0.12	−1.43	0.29	−0.02	−0.34	0.73	−0.05	−0.39	0.70
R frontal	−0.05	−2.231	0.03	−0.07	−2.59	0.01	−0.08	−1.041	0.38	−0.03	−0.50	0.70	−0.03	−0.21	0.83
L temporal	−0.07	−2.967	0.005	−0.06	−2.59	0.01	−0.05	−0.63	0.59	−0.10	−1.52	0.22	0.03	0.17	0.86
R temporal	−0.07	−3.191	0.003	−0.06	−2.65	0.01	−0.04	−0.54	0.59	−0.11	−1.74	0.22	0.02	0.10	0.91
L Occipital	−0.08	−3.166	0.003	−0.04	−1.41	0.14	−0.14	−1.63	0.28	−0.20	−2.80	0.21	0.10	0.60	0.55
R Occipital	−0.07	−2.871	0.005	−0.02	−0.81	0.42	−0.14	−1.63	0.28	−0.16	−2.26	0.09	0.01	0.07	0.93
L parietal	−0.07	−2.858	0.005	−0.07	−2.53	0.01	−0.12	−1.41	0.28	−0.10	−1.41	0.22	0.08	0.54	0.59
R parietal	−0.09	−3.843	<0.001	−0.07	−2.62	0.01	−0.12	−1.45	0.28	−0.17	−2.54	0.22	0.04	0.27	0.79

*Note*: Bolded values are those that remained significant after correction for multiple comparisons.

Abbreviations: L, left hemisphere; R, right hemisphere; WMH, white matter hyperintensity.

### 3.4 Mediation analysis of education, vascular risk, and WMH burden

**FIGURE 2:**
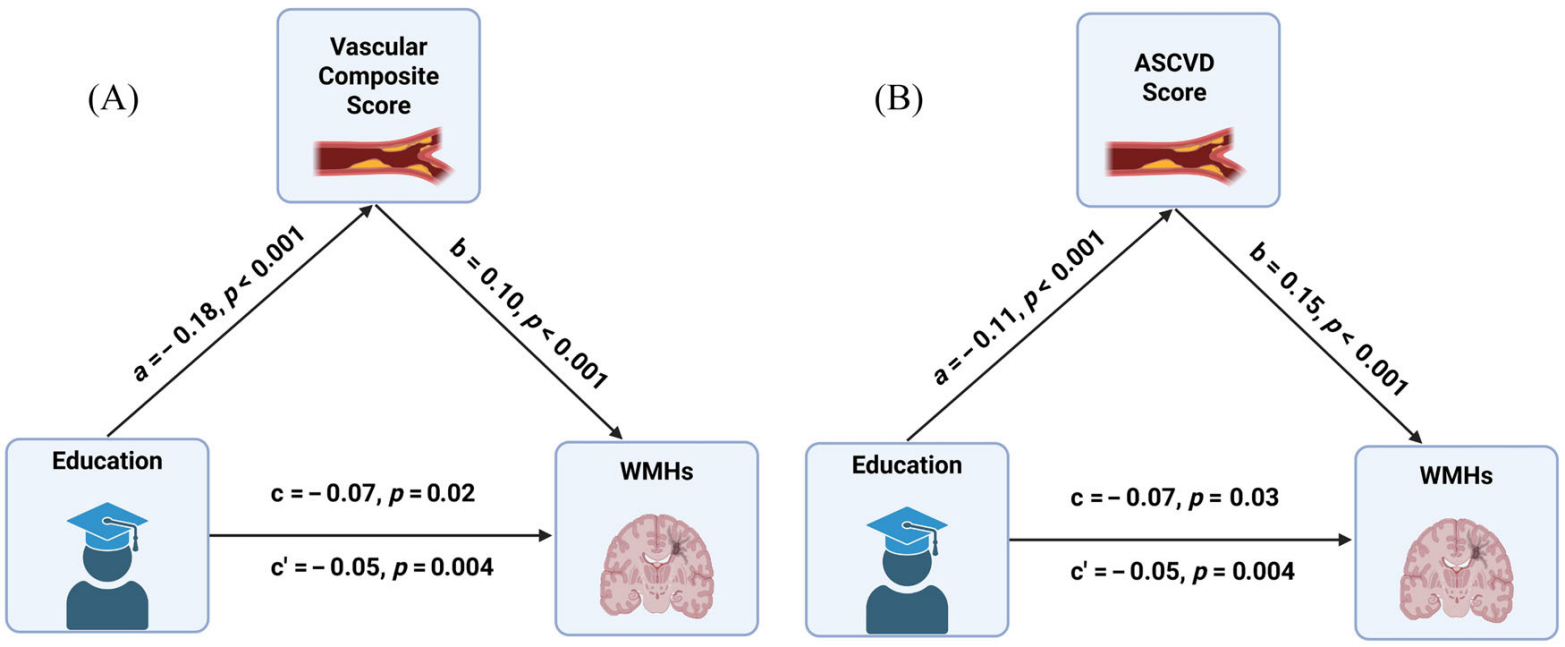
Mediation models illustrating indirect effect of education on total WMH burden through (A) the vascular composite score and (B) the ASCVD BMI‐based score. All path coefficients are standardized and significant (*p* < 0.05). Vascular risk partially mediated the relationship between education and WMH burden, accounting for approximately 27% of the total effect. ASCVD, Atherosclerotic Cardiovascular Disease; BMI, body mass index; WMH, white matter hyperintensity.

Mediation analyses were conducted to determine whether vascular risk factors mediated the relationship between educational attainment and WMH burden. The models assessed both the direct effect of education on WMH volume and the indirect effect through vascular risk (composite and ASCVD BMI‐based scores), adjusting for age, sex, race, and diagnosis (Figure 2).

#### 3.4.1 Mediation using vascular composite score

Across the full sample, vascular risk significantly mediated the association between education and total WMH burden (indirect effect = –0.02, *p* < 0.001; 27% mediated; Table 4). The direct effect of education remained modest but significant (*β* = –0.05, *p* = 0.004). The results were consistent in the model without covariates (indirect effect = –0.03, *p* < 0.001). Region‐specific analyses indicated that education was inversely related to WMH burden across all lobes, but the strength and significance of mediation varied by region. Indirect effects through vascular risk reached statistical significance in several regions (*p* ≤ 0.01), whereas the proportions mediated did not. The largest proportions of indirect effects were observed in the left frontal, right frontal, and left temporal regions (28% to 47%), although these did not survive correction for multiple comparisons.

**TABLE 4:** Mediation analysis (direct and indirect effects).

Region	Composite Score (b)	*P* value (b)	Direct Effect (c’)	*P* value (c’)	Indirect effect (a*b)	*P* value (a*b)	Total effect (c)	*P* value (c)	Proportion mediated (%)	*P* value (c)
**Composite vascular risk score**										
Total WMH	0.10	**<0.001**	−0.05	**0.004**	−0.02	**<0.001**	−0.07	**0.02**	27	**0.03**
L frontal	0.12	**<0.001**	−0.02	0.39	−0.02	**<0.001**	−0.05	0.11	47	0.12
R frontal	0.10	**<0.001**	−0.04	0.18	−0.02	**<0.001**	−0.06	0.06	32	0.07
L temporal	0.09	**<0.001**	−0.05	0.13	−0.02	**<0.001**	−0.06	**0.04**	28	0.06
R temporal	0.06	**0.006**	−0.05	0.10	−0.01	**0.01**	−0.06	**0.02**	18	0.06
L occipital	0.05	0.05	−0.05	0.10	−0.01	0.05	−0.05	**0.04**	15	0.09
R occipital	0.05	0.07	−0.04	0.18	−0.01	0.09	−0.05	0.09	16	0.16
L parietal	0.08	**<0.001**	−0.05	0.11	−0.02	**<0.001**	−0.07	**0.01**	23	0.06
R parietal	0.05	**0.02**	−0.06	**0.04**	−0.05	**0.04**	−0.08	**0.02**	13	0.07
**ASCVD BMI‐based score**										
Total WMH	0.15	**<0.001**	−0.05	**0.01**	−0.01	**0.003**	−0.07	**0.03**	22	**0.04**
L frontal	0.15	**<0.001**	−0.02	0.29	−0.02	**<0.001**	−0.05	0.08	36	0.08
R frontal	0.14	**<0.001**	−0.04	0.11	−0.01	**<0.001**	−0.05	0.05	25	0.05
L temporal	0.12	**0.005**	−0.05	0.10	−0.01	**0.003**	−0.06	**0.02**	20	**0.03**
R temporal	0.10	**0.006**	−0.05	0.10	−0.01	**0.005**	−0.07	**0.02**	17	**0.04**
L occipital	0.10	**0.02**	−0.05	0.10	−0.01	**0.01**	−0.06	0.05	17	0.05
R occipital	0.12	**0.006**	−0.04	0.20	−0.01	**0.009**	−0.05	0.08	27	0.07
L parietal	0.14	**0.002**	−0.05	0.05	−0.01	**<0.001**	−0.07	**0.02**	21	**0.03**
R parietal	0.12	**0.006**	−0.06	0.05	−0.01	**<0.001**	−0.08	**<0.001**	15	**<0.001**

*Note*: Bolded values are those that remain significant after correction for multiple comparisons.

Abbreviations: ASCVD, Atherosclerotic Cardiovascular Disease risk assessment; BMI, body mass index; L, left hemisphere; R, right hemisphere; WMH, white matter hyperintensity.

#### 3.4.2 Mediation using ASCVD BMI‐based score

When vascular risk was indexed by the ASCVD BMI‐based score, results closely paralleled the composite‐score models (Table 4). For total WMH volume, the indirect effect was significant (*β* = −0.01, *p* = 0.003), mediating 22% of the total association (*c* = −0.07, *p* = 0.03). Regionally, indirect effects were significant across all lobes (*β* ≈ −0.01 to −0.02, *p* ≤ 0.01), with the proportion mediated spanning 15% to 36%. The results were consistent in the model without covariates, with a larger indirect effect for total WMH (*β* = −0.03, *p* < 0.001), 25% proportion mediated, and a stronger total effect (*c* = −0.12, *p* < 0.001). No significant interaction effects (Education × Race or Vascular Risk × Race) were detected, indicating that the direction of mediation was consistent across racial groups, although effect magnitudes varied.

#### 3.4.3 Race‐stratified mediation analyses

These analyses indicate that vascular risk primarily among White participants partially mediates education‐related reductions in WMH burden, while statistical power and sample sizes limit conclusive findings for other racial groups. When the analyses were repeated separately within each racial group (Table S1), significant indirect effects were observed only among White participants, where the composite vascular risk score showed a significant mediation effect (*β* = –0.02, *p* = 0.01), accounting for approximately 23% of the total association (*c* = –0.07, *p* = 0.04). When vascular risk was indexed by the ASCVD BMI‐based score, the indirect effect among White participants was smaller (*β* = –0.01, *p* = 0.05), and the proportion mediated (13%) was not statistically significant, indicating a similar but weaker mediation pattern. Effects were again non‐significant among non‐White participants across both scoring methods.

#### 3.4.4 Single‐mediator and leave‐one‐out analyses

To determine which vascular risk factors contributed most to the overall mediation effect, single‐mediator models were used (Table 5). No single variable fully explained the mediation pathway between education and WMH burden, as none of the individual vascular risk factors showed a statistically significant indirect effect. The strongest but modest indirect effect (*β* = –0.01, *p* = 0.01) was observed in smoking duration (years), which accounted for about 22% of the total effect, though the proportional effect was not statistically significant. The largest proportional mediated effect was also observed for smoking duration, while the largest direct effects were seen for systolic and diastolic blood pressure (*β* = −0.07, *p* = 0.04 for both). These findings suggest that the mediation represents a cumulative vascular influence rather than the effect of any single factor.

**TABLE 5:** Single‐mediator analyses of individual vascular risk factors.

	Direct effect (c’)	*P* value (c’)	Indirect effect (a*b)	*P* value (a*b)	Total effect (c)	*P* value (c)	Proportion mediated (%)	*P* value (c)
Systolic blood pressure	−0.07	**0.04**	0.00	0.15	−0.07	**0.04**	4	0.18
Diastolic blood pressure	−0.07	**0.04**	0.00	0.31	−0.07	**0.04**	2	0.33
BMI	−0.05	0.05	0.00	0.54	−0.06	0.05	4	0.51
Smoking (years)	−0.04	0.03	−0.01	**0.01**	−0.05	0.12	22	0.13
Smoking (packs per day)	−0.05	0.13	−0.01	0.06	−0.05	0.08	14	0.13

*Note*: Bolded values are those that remain significant after correction for multiple comparisons.

Abbreviation: BMI, body mass index.

The robustness of the composite mediation model was further validated by leave‐one‐out analyses (Table S2). Regardless of which particular risk factor (including diabetes, hypertension, smoking, or BMI) was eliminated from the composite score, the overall indirect effect remained significant (indirect ≈ –0.02, *p* ≤ 0.002). Excluding diastolic blood pressure contributed to the largest mediated proportion of the total effect (37%). These results again suggest that multiple vascular pathways, rather than being driven by a single dominant factor, jointly contribute to the education–WMH relationship.

In summary, the association between education and both total and regional WMH burden was partially mediated by vascular risk factors collectively, but not by individual factors, accounting for approximately 20% to 30% of the total effect. This mediation was consistent across both weighted and unweighted (ASCVD BMI‐based) indices.

## 4 DISCUSSION

This study investigated whether vascular risk factors mediated the relationship between educational attainment and WMH burden in older adults. Our findings demonstrate that higher education is significantly associated with reduced WMH burden across total and regional brain areas, with associations observed across all lobes (frontal, temporal, parietal, and occipital) and the largest effect sizes in the parietal and occipital regions. Higher education has consistently been associated with lower dementia risk, slower cognitive decline, and improved resilience to structural brain changes.^18^, ^24^ In the present study, vascular risk partially mediated the education–WMH association, suggesting that part of the protective effect of education may occur through healthier cardiovascular profiles.^58^, ^59^, ^60^ These results reinforce a pathway in which higher education is linked to lower cumulative vascular burden and, consequently, to reduced WMH accumulation.^8^, ^9^, ^18^, ^22^, ^59^

Our results showed that higher education was associated with lower levels of individual vascular risk factors, including diabetes, hypertension, hypercholesterolemia, BMI, and smoking exposure. This finding is consistent with population‐level studies indicating that individuals with higher education are more likely to engage in health‐promoting behaviors and experience better cardiovascular outcomes.^24^, ^28^, ^58^ Consequently, both our unweighted composite vascular score and the ASCVD BMI‐based score were inversely related to education, with slightly stronger associations for the composite score, likely because it captures the cumulative burden of multiple vascular comorbidities and behavioral risk factors that are strongly shaped by educational and socioeconomic influences. In contrast, the ASCVD BMI‐based score places greater weight on age and blood pressure and was originally optimized for predicting 10‐year cardiovascular events in middle‐aged adults,^53^, ^54^, ^55^ making it less sensitive to the educational and lifestyle gradients that characterize older populations. Such behavioral and systemic advantages may contribute to reduced cerebrovascular burden and slower progression of WMHs.^8^, ^9^, ^18^ In our sample, education was not significantly related to diastolic blood pressure, which may reflect the relative stability of diastolic pressure in older adulthood.

Education was inversely associated with WMH burden after adjusting for covariates, including diagnosis. This finding is in line with prior studies demonstrating that education contributes to cerebrovascular integrity and preserves white matter microstructure,^22^, ^24^, ^61^, ^62^ with individuals possessing higher educational attainment showing slower accumulation of WMHs in later life.^63^ Regionally, education–WMH associations were statistically significant across all lobes in the full sample, though the strength varied by region, with the largest effects in parietal and occipital areas and modest but reliable effects in frontal and temporal lobes. These lobar patterns extend previous findings that primarily examined global WMH burden, revealing that education‐related resilience was widespread but regionally heterogeneous. Recent studies showed vascular risk factors were most strongly associated with anterior (frontal‐periventricular) WMHs,^15^, ^50^ while posterior lesions, particularly parietal and occipital, were more closely linked to neurodegenerative and amyloid processes.^18^, ^63^, ^64^, ^65^ Therefore, our observation of stronger education–WMH associations in posterior regions is particularly noteworthy, as posterior WMHs are among the most predictive of cognitive decline and AD progression. The current findings suggest that higher education may buffer the impact of both vascular and neurodegenerative processes. This interpretation supports the “dual‐mechanism” theory, indicating education promotes protection in the parts of the brain most susceptible to dementia‐related pathology, particularly the posterior regions impacted by neurodegenerative processes, while also enhancing generalized vascular resilience.^19^, ^60^, ^61^, ^65^, ^66^

In the race‐stratified models, the associations were most stable in White participants, whereas estimates in non‐White subgroups did not consistently reach significance, patterns we attribute to smaller subgroup sizes and reduced statistical power in these groups. Sufficiently powered studies within larger non‐White groups are thus warranted to establish potential differences across racial and ethnic groups.

Mediation analysis revealed that vascular risk factors partially mediated the relationship between education and WMH burden, accounting for approximately 22% to 27% of the total effect. Results were robust when vascular risk was operationalized as either an unweighted composite index or a weighted ASCVD BMI‐based score and were supported by sensitivity analyses. This finding is also consistent with previous studies that highlighted the dual impact of education on brain health, directly through cognitive reserve and indirectly by promoting healthier vascular profiles.^22^, ^23^, ^66^ Individuals with lower educational attainment might be more likely to engage in unhealthy behaviors (such as smoking and physical inactivity) or have higher rates of vascular conditions like hypertension, diabetes, and obesity compared to those with more education.^61^, ^66^ Our data extend previous observations by showing that vascular risk factors mediate approximately 25% of the association between education and WMH burden. Despite the modest magnitude of the effect, given the multifactorial etiology of WMHs and the high prevalence of vascular risks in aging populations, this finding has important implications. Regionally, indirect effects through vascular risk were variable across lobes; several reached significance, but many proportional‐mediation estimates did not survive multiple‐comparison correction, indicating small, region‐dependent contributions of vascular pathways to education–WMH links. This finding may reflect the heterogeneous etiology of WMHs in different regions.^18^, ^50^, ^64^, ^67^

Single‐mediator models did not identify a single dominant factor; smoking duration showed the largest (still modest) indirect effect, and leave‐one‐out analyses showed that removing any one risk factor did not abolish the overall indirect pathway. This finding underscores a cumulative, multifactorial mechanism: No single risk factor can explain the education effect, but collectively they do. Previous research demonstrated that various vascular risk factors are associated with dementia risk^10^, ^30^, ^49^, ^57^, ^58^ and that their prevalence differs across levels of education.^66^ Our findings, showing that these factors mediate part of the education–WMH relationship, highlight the importance of addressing multiple vascular risks simultaneously to reduce dementia vulnerability later in life, particularly among individuals with lower educational attainment.

Several limitations in the current study warrant consideration. While the cross‐sectional design precludes causal inference, our findings are supported by a biologically plausible temporal ordering (education → vascular risk → WMH). However, longitudinal data with time‐ordered exposures and outcomes is warranted to test causal mediation. Participants in the NACC are volunteers who are often more educated and healthier than the general population, which could limit generalizability. Non‐White subgroup analyses were underpowered. As such, larger, more diverse samples are needed to refine estimates and test moderation hypotheses with adequate precision. Our composite score uses binary components to accommodate data availability within the NACC cohort. While interpretable and additive, this simplification does not capture continuous risk gradients or laboratory biomarkers. The ASCVD BMI‐based score served as a weighted sensitivity index, but its derivation cohort (ages 30 to 74, CVD‐free) differs from our older sample (mean age ∼74) with uncertain vascular histories, which could attenuate its informativeness in this context. Finally, alcohol misuse was measured with a coarse clinical indicator, and ambulatory blood pressure or lipid subfractions were unavailable. Future studies should incorporate richer vascular phenotyping.

In conclusion, our study supports the hypothesis that education influences WMH burden in part by reducing vascular disease burden. From a public health perspective, even small indirect effects matter because education and vascular risks are common exposures. As such, modest shifts in vascular health could translate into meaningful reductions in WMH accumulation and downstream cognitive decline. Clinically, these findings reinforce intensive vascular risk management as relevant not only to cardiovascular outcomes but also to brain health. Meanwhile, education emerges as a lifelong determinant of cerebrovascular integrity, underscoring the importance of early‐life educational equity coupled with sustained vascular risk control in adulthood. Overall, our findings offer a better understanding of how education protects the cerebrovascular system in aging populations and emphasize integrating educational equity and vascular risk prevention in public health strategies to mitigate cerebrovascular disease and slow cognitive aging.

## AUTHOR CONTRIBUTIONS

Shima Raeesi, Roqaie Moqadam, Yashar Zeighami, Mahsa Dadar, and Cassandra Morrison were involved with the conceptualization and design of the work. Shima Raeesi, Yashar Zeighami, and Mahsa Dadar completed the analysis. Cassandra Morrison, Mahsa Dadar, Roqaie Moqadam, Yashar Zeighami, and Shima Raeesi were involved with data interpretation. Shima Raeesi wrote the manuscript. Cassandra Morrison, Roqaie Moqadam, Yashar Zeighami, Mahsa Dadar, and Shima Raeesi revised and approved the submitted version.

## CONFLICT OF INTEREST STATEMENT

The authors declare no conflicts of interest. Author disclosures are available in the Supporting Information.

## CONSENT STATEMENT

Informed consent was obtained in writing from each participant or their designated study partner.

## Supporting information



Supporting Information

Supporting Information

## Data Availability

The data utilized in this study were also sourced from the National Alzheimer's Coordinating Center (NACC) database (https://naccdata.org/), specifically drawing from the NACC UDS and MRI Data Set (Beekly et al., 2004; Besser, Kukull, Knopman, et al., 2018; Besser, Kukull, Teylan, et al., 2018).
