# Supplementary material for: Vascular risk factors mediate the relationship between education and white matter hyperintensities
Source: Alzheimers Dement. 2025 Dec 15;21(12):e70972. doi: 10.1002/alz.70972 (PMC12706122; doi:10.1002/alz.70972)
Supplement: Supplementary file 2 — Supporting Information [file ALZ-21-e70972-s002.docx]

**Table S1: Stratified (within-race) Mediation Analysis**

|  | **Direct Effect (c’)** | ***p* value (c’)** | **Indirect Effect (a*b)** | ***p* value (a*b)** | **Total Effect**  **(c)** | ***p* value (c)** | **Proportion mediated (%)** | ***p* value (c)** |
| --- | --- | --- | --- | --- | --- | --- | --- | --- |
| **Composite vascular risk score** | | | | | | | | |
| White | -0.06 | 0.14 | -0.02 | **0.01** | -0.07 | **0.04** | 23 | **0.03** |
| Hispanic | -0.04 | 0.41 | -0.01 | 0.34 | -0.04 | 0.34 | 13 | 0.55 |
| Black | -0.10 | 0.46 | -0.03 | 0.27 | -0.13 | 0.39 | 22 | 0.48 |
| Asian | - | - | - | - | - | - | - | - |
| **ASCVD BMI-based score** | | | | | | | | |
| White | -0.07 | 0.11 | -0.01 | 0.05 | -0.08 | 0.05 | 13 | 0.13 |
| Hispanic | -0.02 | 0.71 | -0.02 | 0.06 | -0.04 | 0.41 | 47 | 0.46 |
| Black | -0.12 | 0.40 | -0.04 | 0.08 | -0.15 | 0.31 | 24 | 0.33 |
| Asian | - | - | - | - | - | - | - | - |

*ASCVD = Atherosclerotic CardioVascular Disease risk assessment. BMI = Body Mass Index. Bolded values are*

*those that remain significant after correction for multiple comparisons.*

**Table S2: Leave-One-Out Sensitivity Analyses of the Composite Vascular Risk Model in the Total Sample**

|  | **Direct Effect (c’)** | ***p* value (c’)** | **Indirect Effect (a*b)** | ***p* value (a*b)** | **Total Effect**  **(c)** | ***p* value (c)** | **Proportion mediated (%)** | ***p* value (c)** |
| --- | --- | --- | --- | --- | --- | --- | --- | --- |
| Diabetes | -0.05 | 0.08 | -0.01 | **0.002** | -0.07 | **0.03** | 20 | **0.03** |
| Hypertension | -0.06 | 0.07 | -0.02 | **<0.001** | -0.07 | **0.03** | 16 | **0.04** |
| Systolic Blood Pressure | -0.05 | 0.11 | -0.02 | **<0.001** | -0.07 | **0.03** | 28 | **0.03** |
| Diastolic Blood Pressure | -0.04 | 0.15 | -0.02 | **<0.001** | -0.07 | **0.03** | 37 | **0.03** |
| Hypercholesterolemia | -0.05 | 0.08 | -0.01 | **0.002** | -0.07 | **0.03** | 21 | **0.03** |
| Alcohol Abuse | -0.05 | 0.10 | -0.02 | **<0.001** | -0.07 | **0.03** | 26 | **0.03** |
| Smoking | -0.05 | 0.09 | -0.02 | **<0.001** | -0.07 | **0.03** | 27 | **0.03** |
| BMI | -0.05 | 0.10 | -0.02 | **<0.001** | -0.07 | **0.03** | 27 | **0.03** |
| Composite Score (8) | -0.05 | 0.10 | -0.02 | **<0.001** | -0.07 | **0.03** | 27 | **0.03** |

*BMI = Body Mass Index. Bolded values are those that remain significant after correction for multiple comparisons.*
